# Immunohistochemical Characterisation of the Whale Retina

**DOI:** 10.3389/fnana.2022.813369

**Published:** 2022-02-04

**Authors:** Noelia Ruzafa, Xandra Pereiro, Elena Vecino

**Affiliations:** ^1^Experimental Ophthalmo-Biology Group (GOBE), Department of Cell Biology and Histology, University of Basque Country UPV/EHU, Leioa, Spain; ^2^Begiker-Ophthalmology Research Group, Biocruces Health Research Institute, Cruces Hospital, Bilbao, Spain

**Keywords:** retina, whale, cetacean, visual system, evolutionary neuroscience, glia, neuron, retinal ganglion cell (RGC)

## Abstract

The eye of the largest adult mammal in the world, the whale, offers a unique opportunity to study the evolution of the visual system and its adaptation to aquatic environments. However, the difficulties in obtaining cetacean samples mean these animals have been poorly studied. Thus, the aim of this study was to characterise the different neurons and glial cells in the whale retina by immunohistochemistry using a range of molecular markers. The whale retinal neurons were analysed using different antibodies, labelling retinal ganglion cells (RGCs), photoreceptors, bipolar and amacrine cells. Finally, glial cells were also labelled, including astrocytes, Müller cells and microglia. Thioflavin S was also used to label oligomers and plaques of misfolded proteins. Molecular markers were used to label the specific structures in the whale retinas, as in terrestrial mammalian retinas. However, unlike the retina of most land mammals, whale cones do not express the cone markers used. It is important to highlight the large size of whale RGCs. All the neurofilament (NF) antibodies used labelled whale RGCs, but not all RGCs were labelled by all the NF antibodies used, as it occurs in the porcine and human retina. It is also noteworthy that intrinsically photosensitive RGCs, labelled with melanopsin, form an extraordinary network in the whale retina. The M1, M2, and M3 subtypes of melanopsin positive-cells were detected. Degenerative neurite beading was observed on RGC axons and dendrites when the retina was analysed 48 h post-mortem. In addition, there was a weak Thioflavin S labelling at the edges of some RGCs in a punctuate pattern that possibly reflects an early sign of neurodegeneration. In conclusion, the whale retina differs from that of terrestrial mammals. Their monochromatic rod vision due to the evolutionary loss of cone photoreceptors and the well-developed melanopsin-positive RGC network could, in part, explain the visual perception of these mammals in the deep sea.

## Introduction

Cetaceans are a mammalian group that contains some of the largest animals on Earth and they offer a unique opportunity to investigate the adaptations of the visual system to the aquatic environment. However, despite its importance, the cetacean visual system has been little studied, and the morphological and anatomical characteristics of the eyes and retina of various cetacean species remain completely unknown. We assume that studying the structure and morphological features of the retina of these large mammals may help us to understand the adaptation of their vision to the deep sea.

The analysis of retinal ganglion cells (RGCs), the neurons that transmit visual information from the retina to the brain, has been analysed in a variety of cetacean species in order to estimate their visual acuity. The visual acuity appear to be similar in most marine cetaceans and the retinal resolving power is lower than in terrestrial mammals (Murayama et al., 1992; Murayama and Somiya, 1998). Moreover, it is noteworthy to point out that the RGCs named giant cells, the cell body reaches up to 75 μm in diameter (Dawson et al., 1982; Mengual et al., 2015). Due to the enormous size of these cetaceans, their giant RGCs and their very long axons, it was deemed necessary to analyse the neurofilament (NF) expression in the RGCs. The NFs are the predominant intermediate filament proteins in neurons, and they are assemblies of three subunits that form heteropolymeric filaments that are running along the length of the axon, and in the soma, primarily serving a structural function. Not all neurons have the same combination of NF subunits and there are clear differences between species (Ruiz-Ederra et al., 2004). Here, the expression of different RGC markers, including NFs, has been analysed for the first time in the largest animals in the world.

Melanopsin is the visual pigment expressed by a small subset of RGCs, intrinsically photosensitive retinal ganglion cells (ipRGCs). These ipRGCs are mainly implicated in non-image forming functions such as regulation of circadian rhythms or activation of the pupillary light reflex (Lax et al., 2019). Genetic analyses, coupled with molecular modelling, predict that cetacean melanopsin possesses a nearly identical absorption spectra to that of terrestrial mammals (Fasick and Robinson, 2016). However, relatively little is known of the role of melanopsin and ipRGCs in cetaceans.

In addition to RGCs, photoreceptors respond to light, and they have also been studied in cetacean retinas. Most terrestrial mammals have colour vision based on the different visual pigments in the cone photoreceptors. Among terrestrial mammals, the absence of cone-based colour vision is rare and is generally restricted to nocturnal animals. However, a range of marine mammals have consistently demonstrated the absence of S-cones, including toothed whales. However, unlike the terrestrial cone monochromats, these marine mammals have phases of daylight activity. Thus, the evolutionary convergent loss of S-cones in marine species may offer an adaptive advantage in the marine environment (Peichl et al., 2001).

Understanding the visual system of cetaceans is of great interest in comparative anatomy and physiology. Thus, here we present a complete morphological analysis of the retina of two Balaenoptera species. In addition to studying their RGCs and photoreceptors, other retinal cells were also analysed, including ipRGCs, as well as bipolar and amacrine cells. In addition, the glial cells that support and protect the retinal neurons were also analysed, including astrocytes, Müller cells, and microglia. In addition, rat and pig retinas were used to compare the whale retina with that of terrestrial mammals to obtain a more complete characterisation of cetacean retinas.

## Materials and Methods

### Eye Samples and Tissue Collection

The eyes of beached *Balaenoptera physalus* and *Balaenoptera borealis* whales (*n* = 2, one eye from each whale), were extracted and studied for 24 and 48 h post-mortem, respectively. The ocular globes, after extraocular muscles were removed, weigh approximately 1 kg. The eyes were cut down to half and were fixed overnight in 4% paraformaldehyde (PFA) prepared in 0.1 M phosphate buffer (PB, pH 7.4) at 4°C. The retinas were extracted after fixation. Most of the results presented here were obtained from *B. physalus* retina, which was in better condition, although these results were corroborated with the *B. borealis* retina.

**FIGURE 1:**
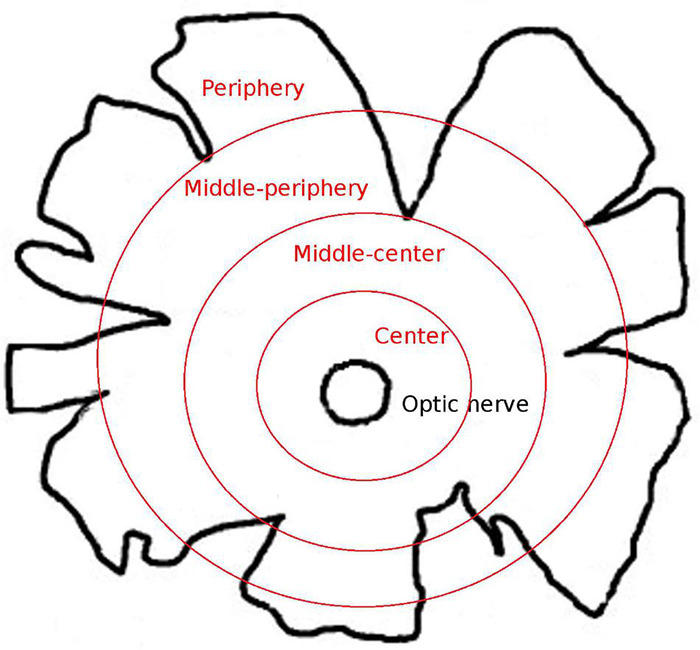
Scheme of the whale retinal areas. Scheme of a whale retina in which the four different areas of the retina are represented as concentric circles in red. The areas were classified based on their distance from the optic nerve: center, a 2-cm thick band near the optic nerve; middle-center, a 2.5-cm thick band outside the center band; middle-periphery, a 2.5-cm thick band adjacent to the middle-center band; and periphery, a 2-cm thick band at the edge of the retina and the furthest from the optic nerve.

The whale retinas were divided into four different disc shaped areas at different distances from the optic nerve: centre (a 2-cm thick band near the optic nerve), middle-centre (a 2.5 cm thick band outside the centre band), middle-periphery (a 2.5 cm thick band adjacent to the middle-centre band), and periphery (a 2 cm thick band at the edge of the retina and the furthest from the optic nerve; Figure 1). At least three pieces of each region that were taken along the dorsal axis were analysed. The different retinal regions were then cut into pieces of approximately 1 cm^2^ for immunostaining and analysis. To obtain the sections, the retinal tissue, from the four retinal areas described in Figure 1, was cryoprotected for 24 h in 30% sucrose in 0.1 M PB at 4°C and embedded in Tissue-Tek O.C.T. compound (Sakura, Netherlands), and the cryosections (14 μm thick) were then stored at −20°C.

Adult porcine eyes were obtained from a local slaughterhouse and transported to the laboratory in cold CO_2_-independent medium (Life Technologies, Carlsbad, CA, United States) plus 0.1% gentamicin. Adult Sprague Dawley rat eyes were obtained from animals that were housed under a 12-h light–dark cycle with *ad libitum* access to food and water and were humanely sacrificed by exposure to CO_2_. To obtain the whole-mount retina, the eyes were dissected, the entire retina was isolated, and the retinas were fixed in 4% PFA. To obtain the sections, whole eyes were fixed in 4% PFA overnight and cryoprotected for 24 h in 30% sucrose in 0.1 M phosphate buffer at 4°C, finally the eyes were embedded in OCT medium. Cryosections (14 μm thick) were obtained and stored at –20°C.

Animal experimentation adhered to the association for research in vision and ophthalmology (ARVO) Statement for the Use of Animals in Ophthalmic and Vision Research. Moreover, all the experimental protocols complied with the European (2010/63/UE) and Spanish (RD53/2013) regulations regarding the protection of experimental animals, and they were approved by the Ethics Committee for Animal Welfare at the University of the Basque Country.

### Immunochemistry and Image Capture

Whole mount retinas were immunostained as described previously (Ruzafa et al., 2018), with minor modifications. The flat fixed retinal tissue was washed in phosphate-buffered saline (PBS, pH 7.4) and non-specific binding was blocked by incubating them overnight at 4°C with shaking in a solution of PBS-Tx + BSA (0.25% Triton-X 100 and 1% bovine serum albumin in PBS). The retina was then incubated for 1 day at 4°C with the primary antibodies (Table 1) diluted in PBS-Tx + BSA, after which they were washed three times in PBS for 15 min, and antibody binding was detected over 5 h at room temperature (RT) with shaking using secondary antibodies diluted 1:1,000 in PBS + BSA (1%): Alexa Fluor 555 or 488 conjugated goat anti-mouse, goat anti-rabbit, or donkey anti-goat antibodies (Invitrogen, Eugene, OR, United States). Finally, the retinal tissue was washed three times for 10 min in PBS, flat-mounted onto slides in PBS:glycerol (1:1) and cover slipped.

**TABLE 1:** Primary antibodies used.

Antigen	Target	Host	Dilution	Supplier
βIII Tubulin	RGCs	Mouse	1:2,000	Promega
Brn3a	RGCs	Goat	1:1,000	Santa Cruz Biotechnology
Calbindin	Amacrine cells	Mouse	1:1,000	Swant
Calretinin	Amacrine cells	Rabbit	1:1,000	Sigma
CRALBP	Müller cells	Rabbit	1:1,000	Abcam
GFAP	Astrocytes	Mouse	1:1,000	Sigma
Glutamine synthetase	Müller cells	Rabbit	1:10,000	Abcam
Human Cone arrestin	Cones	Rabbit	1:10,000	William Beltran’s Lab
Iba-1	Microglia	Rabbit	1:2,000	Wako
Melanopsin	ipRGCs	Rabbit	1:1,000	Thermo Scientific
NF-L	RGCs	Rabbit	1:5,000	Sigma
NF-M	RGCs	Mouse	1:1,000	Sigma
NF-H	RGCs	Rabbit	1:500	Sigma
NF-HP	RGCs	Mouse	1:500	Merck Millipore
Opsin S	Cones (blue)	Rabbit	1:1,000	Merck Millipore
Opsin M/L	Cones (red/green)	Rabbit	1:1,000	Merck Millipore
p75NTR	Müller cells	Rabbit	1:2,000	Abcam
PKC-α	Bipolar cells	Mouse	1:2,000	Santa cruz Biotechnology
Rat Cone arrestin	Cones	Rabbit	1:1,000	Merck Millipore
RBPMS	RGCs	Rabbit	1:4,000	PhosphoSolutions
Rhodopsin	Rods	Mouse	1:1,000	Abcam
S100	Müller cells	Rabbit	1:500	Dako
Vimentin	Müller cells	Mouse	1:10,000	Dako

Retinal cryostat sections were immunostained as described previously (Ruzafa et al., 2017). The sections were washed twice with PBS-Tx for 10 min, and then incubated overnight with primary antibodies diluted in PBS-Tx + BSA. After two washes with PBS, antibody binding was detected for 1 h with aforementioned secondary antibodies (Invitrogen) diluted 1:1,000 in PBS + BSA (1%). The sections were then washed twice with PBS for 10 min, mounted in PBS: glycerol (1:1), and cover slipped.

Thioflavin S (ThS) staining was also performed on these retinas after their incubation with the secondary antibodies. The ThS (Sigma–Aldrich, St. Louis, MO, United States) was kindly provided by Estibaliz Capetillo, and it was diluted 1:250 in PB (pH 7.4) and filtered. The retinas were incubated with ThS for 15 min and then washed three times with PB for 5 min with shaking. Finally, the retinas were washed three times with PBS for 10 min, mounted on slides in PBS: glycerol (1:1) and cover slipped.

Images were acquired with a digital camera (Zeiss Axiocam MRM, Zeiss, Jena, Germany), coupled to an epifluorescence microscope (Zeiss) using the Zen software (Zeiss). Approximately 10X and 20X objectives were used. For cell quantification, the contour of the retina was measured and the retinal surface area was calculated using an automated multi-image acquisition using a motorised microscope stage and the Zen software. In the same piece of retina, at least three different areas between 10 and 15 mm^2^ were analysed and different pieces of retina for each region were used. The number of RGCs was counted manually and the cell density was calculated (RGCs/mm^2^, represented as the mean of the different analysed areas). The total retinal area was calculated measuring the retina after its extraction and the total RGC density was estimated after the quantified RGC density in different areas. In addition, the longest diameter of RGCs was measured using the Zen software, after taking pictures of at least 25 RGCs from each region. In order to quantify the different subtypes of melanopsin, whole-mount retinas of RGCs were used, the fine adjustment knob of the microscope was continually used during the manual quantification in order to determine the dendrites stratification of these cells. For that, RNA-binding protein with multiple splicing (RBPMS) labelling was used as reference for the ganglion cell layer (GCL).

## Results

### Retinal Ganglion Cell Analysis

**FIGURE 2:**
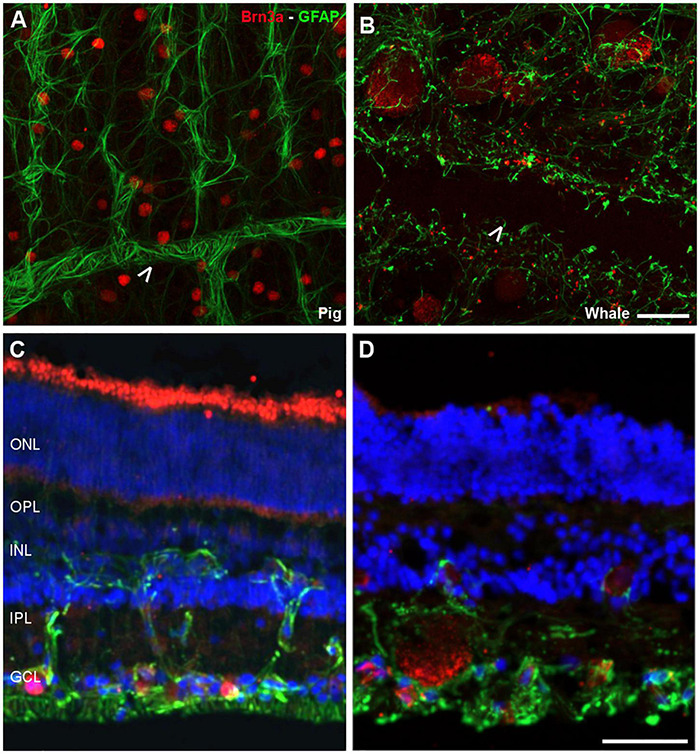
Retinal ganglion cell (RGCs) and astrocytes in the pig and fin whale retina. The cytoskeleton of astrocytes (green) was labelled with an antibody against GFAP and the nuclei of the RGCs (red) with an antibody against Brn3a in both pig **(A,C)** and fin whale **(B,D)** retinas. Whole mount retinas **(A,B)** and sections **(C,D)** are shown. Whale astrocytes do not completely surround the vessels in the whale retina as they do in pigs (white arrowheads **A,B**). The nuclei in the retinal sections were labelled with DAPI (blue **C,D**): ONL, outer nuclear layer; OPL, outer plexiform layer; INL, inner nuclear layer; IPL, inner nuclear layer; GCL, ganglion cell layer. Scale bar = 50 μm.

**FIGURE 3:**
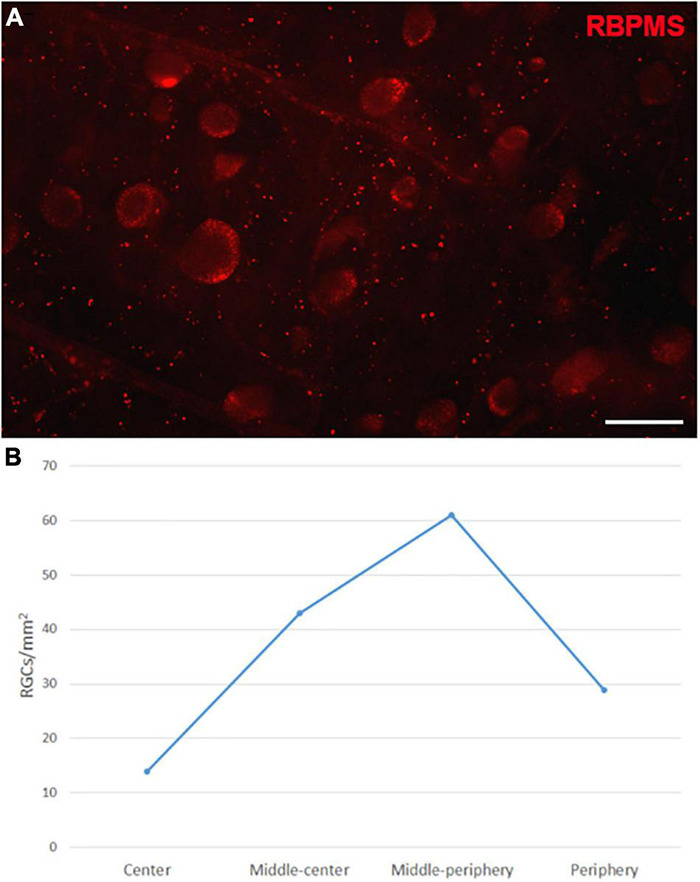
Retinal ganglion cell quantification in the fin whale retina. Image of a whole mount whale retina labelled with an antibody against RBPMS (red) to label RGCs **(A)**. The densities of RGCs (RGCs/mm^2^) in different regions of the whale retina are shown in a histogram **(B)**. Scale bar = 100 μm.

The Retinal Ganglion Cells (RGCs) in the whale retinas were studied by labelling them with specific antibodies. Whale RGCs were labelled for βIII tubulin, Brn3a, RBPMS, and NFs. It is remarkable that the Brn3a labelling in the whale RGCs was not restricted to the nucleus, as the standard labelling pattern of this marker. In whale RGCs, Brn3a staining was more diffused, and it spread into the soma (see Figure 2). A combination of different molecular markers were used to the quantification of RGCs, in particular, the different subunits of NFs and RBPMS. The density of the RGCs in the retina was 36 RGCs/mm^2^. The highest RGC density was detected in the middle-periphery of the retina (61 RGCs/mm^2^) and the lowest concentration of RGCs was in the center of the retina, close to the optic nerve (14 RGCs/mm^2^: Figure 3). Moreover, the total number of RGCs in the fin whale retina was estimated based on the RGC density and the total area of the whale retina (8,800 mm^2^), giving an estimated total number of RGCs in the fin whale retina of 3,25,000 (Table 2). In addition, it is important to note that the large size of the whale RGCs were between 26.5 and 112.9 μm in diameter, with an average size of 52.5 μm in the center and 65.3 μm in the periphery of the retina, reflecting a mild trend in the increase of the soma size toward periphery.

**TABLE 2:** Comparison of retinal ganglion cells (RGCs) analysis published for whales and human retinas.

Species	RGC density (cells/mm^2^)	Total number of RGCs	Retinal area (mm^2^)	References
Pilot whale	267.3	203,000	–	Mengual et al., 2015
Grey whale	70	174,000	2,520	Mass, 1996
Killer whale	290	199,000	1,655	Mass et al., 2013
Fin whale	14–61	325,000	8,800	Present study
Human	32,000–38,000	700,000–1,500,000	1,094	Watson, 2014

**FIGURE 4:**
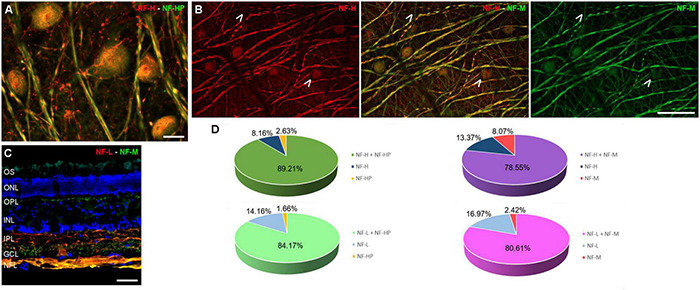
Neurofilament analysis of RGCs in the fin whale retina. Images of whole mount whale retinas **(A,B)**, and retinal sections **(C)** labelled with antibodies against different types of neurofilaments: NF-H (red) and NH-HP (green) **(A)**; NF-H (red) and NH-M (green) **(B)**; and NF-L (red), NH-M (green), and DAPI **(C)**. Note that each type of NF has a specific labelling pattern and not all the neuron structures were labelled with each NF (arrows). The percentage of RGCs labelled with each type of NF was quantified **(D)**. Scale bar = 50 μm.

The RGC subtypes were also analysed using different antibodies against NFs: heavy (H), heavy phosphorylated (HP), medium (M), and low (L). The NFs were distributed widely within the RGC soma, dendrites, and axons, although in some cells NF-H and NF-M staining was more intense in the perinuclear area. Some RGCs do not express all types of NFs, yet a high proportion of RGCs were labelled for at least two NF subunits. The RGCs labelled with antibodies against each type of NF were quantified and the percentage of RGCs were represented (Figure 4 and Supplementary Figure 1A).

### Intrinsically Photosensitive Retinal Ganglion Cells Analysis

**FIGURE 5:**
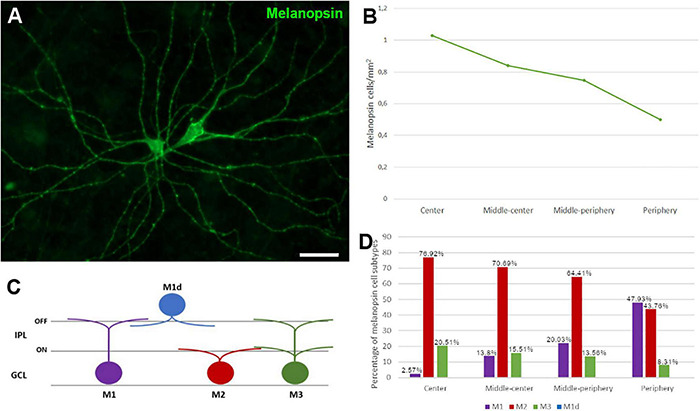
Melanopsin positive cell quantification and classification in the fin whale retina. Melanopsin positive RGCs (green) in the whole mount whale retina **(A)**. Histogram of the total number of melanopsin positive cells/mm^2^ in different regions of the whale retina **(B)**. Melanopsin cells can be classified based on their soma location, typically in the ganglion cell layer (GCL), while their dendrites stratify in the inner plexiform layer (IPL) **(C)**. The proportion of M1, M2, and M3 subtypes of melanopsin positive cells, and their distribution is represented in a histogram **(D)**. Scale bar = 100 μm.

**FIGURE 6:**
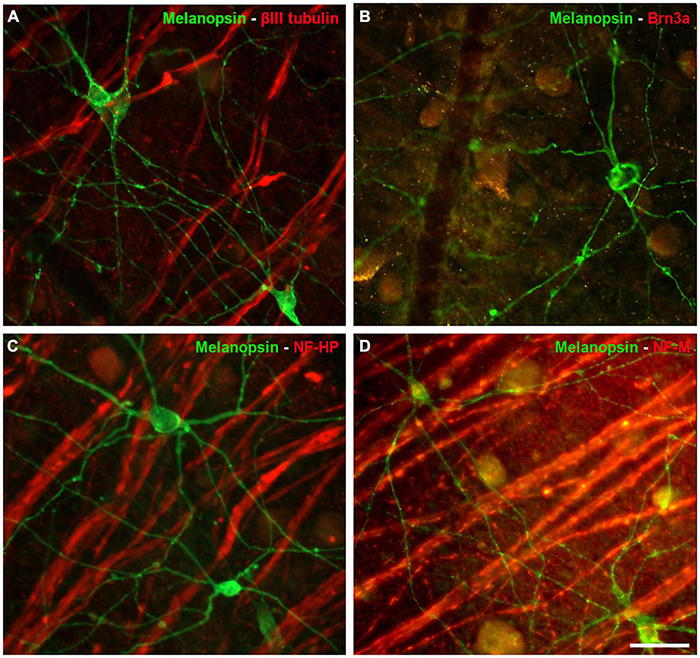
Other RGC markers expressed by Melanopsin positive cells in the whale retina. Images of whole mount whale retinas labelled with antibodies against melanopsin (green) and other specific RGC markers (red): βIII tubulin **(A)**, Brn3a **(B)**, NF-HP **(C)**, and NH-M **(D)**. Note that the melanopsin positive ipRGCs, were not stained with any of the other markers. Scale bar = 100 μm.

The ipRGCs in the retina were labelled with an antibody against melanopsin and these cells formed an extraordinary network covering the surface of the whale retina. The ipRGCs were more abundant in the center of the retina, at a density of 1.03 melanopsin positive cells/mm^2^ close to the optic nerve, which diminished to 0.5 cells/mm^2^ toward the periphery (Figure 5B and Supplementary Figure 1B). In addition, melanopsin cells can be classified depending on the location of their soma and their dendrite stratification. M1 cells have their soma located in the ganglion cell layer (GCL) and their dendrites stratified in the outermost layer of the inner plexiform layer (IPL). The M2 cells have their cell body in the GCL and their dendrites stratified in the innermost layer of the IPL, close to the GCL. The M3 cells have their cell body in the GCL and with dendrites stratified in both the outermost and innermost layer of the IPL. The M1, M2, and M3 melanopsin positive cells were identified in the whale retina (Supplementary Figure 2), although most of them were M2. The proportion of M1 cells increased from the centre to the periphery, whereas the percentage of M2 and M3 cells diminished toward the periphery. The percentage of each subtype of ipRGCs in each retinal region are represented (Figure 5D). Moreover, melanopsin positive ipRGCs were not labelled with other specific molecular markers for RGCs, such as βIII tubulin, Brn3a, or NFs (Figure 6).

### Photoreceptors

**FIGURE 7:**
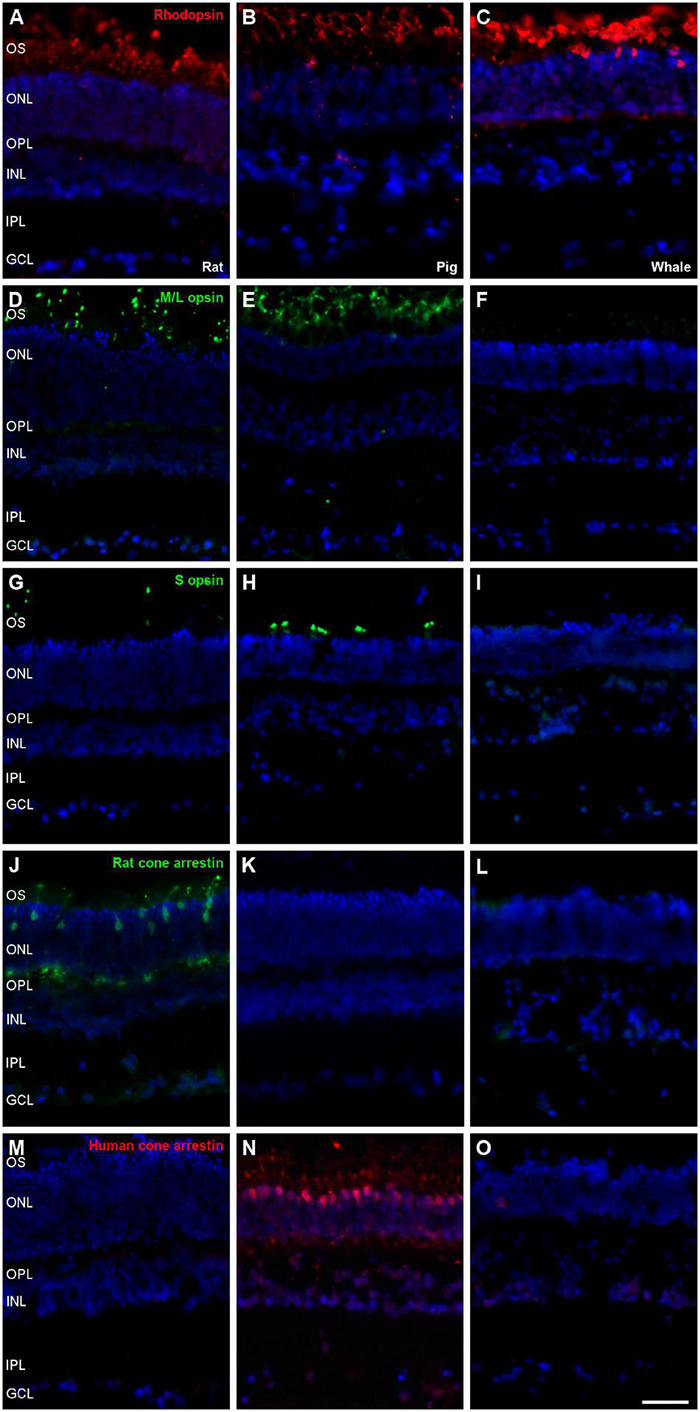
Photoreceptors the in rat, pig and fin whale retina. Sections of rat **(A,D,G,J,M)**, pig **(B,E,H,K,N)**, and whale **(C,F,I,L,O)** retinas. The retinas were labelled with antibodies against rhodopsin (red, **A–C**), M/L opsin (green, **D–F**), S opsin (green, **G–I**), rat cone arrestin (green, **J–L**), and human cone arrestin (red, **M–O**). The nuclei were labelled with DAPI (blue): OS, outer segment; ONL, outer nuclear layer; OPL, outer plexiform layer; INL, inner nuclear layer; IPL, inner nuclear layer; GCL, ganglion cell layer. Scale bar = 50μm.

Photoreceptors were labelled using different antibodies, such as rhodopsin for rods and M/L opsin, S opsin, rat cone arrestin, and human cone arrestin for cones. Rat and pig retinas were used to check the antibody labelling. Rods from the three species were stained with the antibody against rhodopsin, yet unlike the rat and porcine retina, whale cones did not express any of the cone markers (Figure 7). Labelling for M/L and S opsin was observed in the rat and pig retina but not in the whale retina. As rat cone arrestin only labelled the rat retina and human cone arrestin only the pig retina, the specificity of this species of the cone arrestin protein, it would mean that we cannot determine if the whale retina expresses arrestin or whether the antibodies used simply fail to recognise whale cone arrestin.

### Bipolar and Amacrine Cells

**FIGURE 8:**
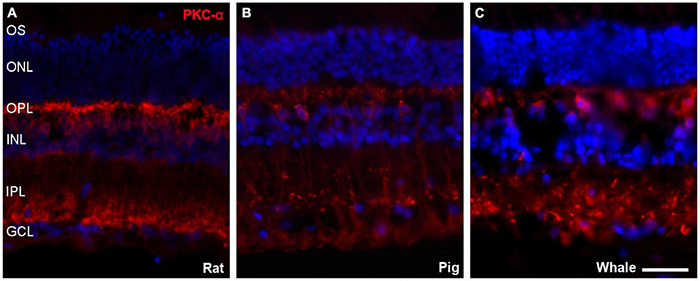
Bipolar cells in the rat, pig, and fin whale retina. Sections from rat **(A)**, pig **(B)**, and whale **(C)** retinas labelled with an antibody against PKC-α (red) to identify bipolar cells. The nuclei were stained with DAPI (blue): OS, outer segment; ONL, outer nuclear layer; OPL, outer plexiform layer; INL, inner nuclear layer; IPL, inner nuclear layer; GCL, ganglion cell layer. Scale bar = 50 μm.

Bipolar cells were also studied in the whale retinas through the abundant expression of Protein kinase C α (PKC-α) by rod bipolar cells. The labelling of these cells was similar to that in the rat and pig retina (Figure 8).

**FIGURE 9:**
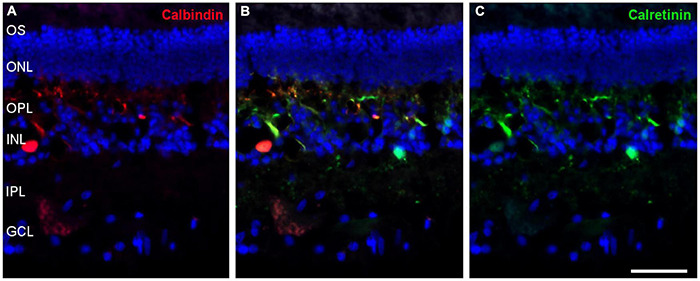
Amacrine cells in the fin whale retina. Sections of whale retinas labelled with antibodies against calbindin (red, **A**) and calretinin (green, **C**) to stain amacrine cells. The merged image of the two antibodies is also shown in panel **(B)**. The nuclei were stained with DAPI (blue): OS, outer segment; ONL, outer nuclear layer; OPL, outer plexiform layer; INL, inner nuclear layer; IPL, inner nuclear layer; GCL, ganglion cell layer. Scale bar = 50 μm.

Most amacrine cells contain calretinin, calbindin or both, although other retinal cells may contain one or other protein. Using antibodies against these proteins, amacrine cells were labelled in the whale retina consistent with their distribution in the inner nuclear layer (Figure 9).

### Glial Cells

Glial cells were also analysed in the whale retina, first labelling astrocytes with an antibody against GFAP. We assumed that some Müller cell processes were also stained. Moreover, the relationship between the RGCs and these glial cells was analysed using an antibody against Brn3a to label the nuclei of RGCs. The whale astrocytes adopted a different pattern to other mammalian retinas (e.g., pig astrocytes). The GFAP labelling in the whale retina was punctuate and the astrocytes seemed smaller than in the pig retina. The blood vessels were not completely surrounded by astrocytes, as occurs in other mammalian retinas (Figure 2). This lack of labelling could be due to the large size of the blood vessels; thus, the focus plane of image is in the lumen of the vessel. However, the astrocytes network that surround the blood vessels in the pig retina seems to be more complex than in the whale retina.

**FIGURE 10:**
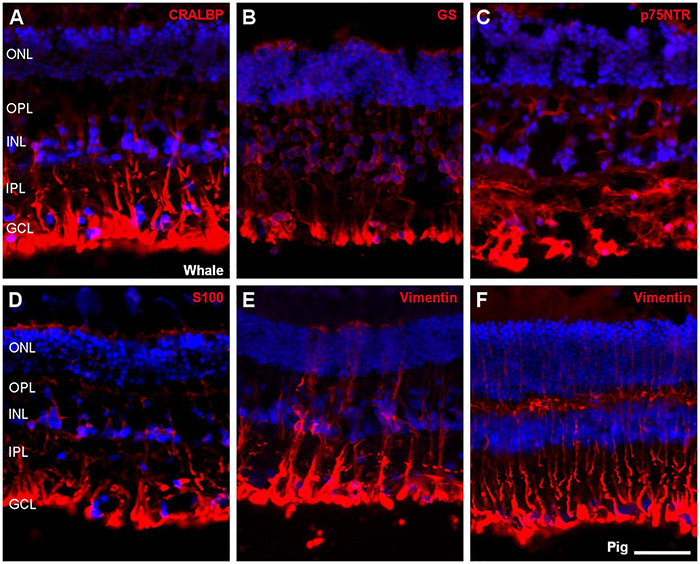
Müller cells in the fin whale and pig retina. Sections from whale retinas labelled with different antibodies to label Müller cells: CRALBP **(A)**, glutamine synthetase (GS, **B**), p75NTR **(C)**, S100 **(D)**, and vimentin **(E)**. A section of the pig retina was also labelled with vimentin **(F)**. The nuclei were stained with DAPI (blue): ONL, outer nuclear layer; OPL, outer plexiform layer; INL, inner nuclear layer; IPL, inner nuclear layer; GCL, ganglion cell layer. Scale bar = 50 μm.

Müller cells were also studied using different antibodies specific to these cells, such as CRALBP, glutamine synthetase (GS), p75NTR, S100, and vimentin. All markers were expressed in the whale Müller cells, which were characteristically radially oriented cells that traverse the retina. In comparison with pig Müller cells, whale Müller cells appeared more robust with thicker end-feet (Figure 10).

**FIGURE 11:**
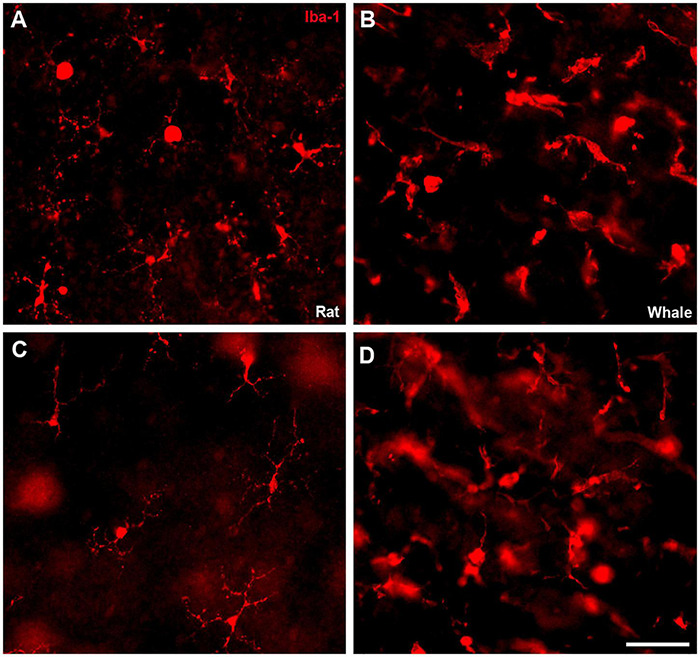
Microglia in the rat and fin whale retina. Microglial cells were labelled with an antibody against Iba1 (red) in rat **(A,C)** and fin whale **(B,D)** whole mount retinas. Images of the microglia were taken close to the ganglion cell layer **(A,B)** and to the inner nuclear layer **(C,D)** in the whole mount retinas. Scale bar = 50 μm.

Microglial cells were analysed in the whale retina using an antibody against Iba1, and assessing their morphology in whole mount retinas at different levels. When the whale retina was compared with the rat retina, whale microglial cells seemed to be more abundant and larger in size. In addition, the limits of the microglial cells were difficult to identify in comparison with rat microglial cells (Figure 11).

### Neurodegeneration in the Whale Retina

**FIGURE 12:**
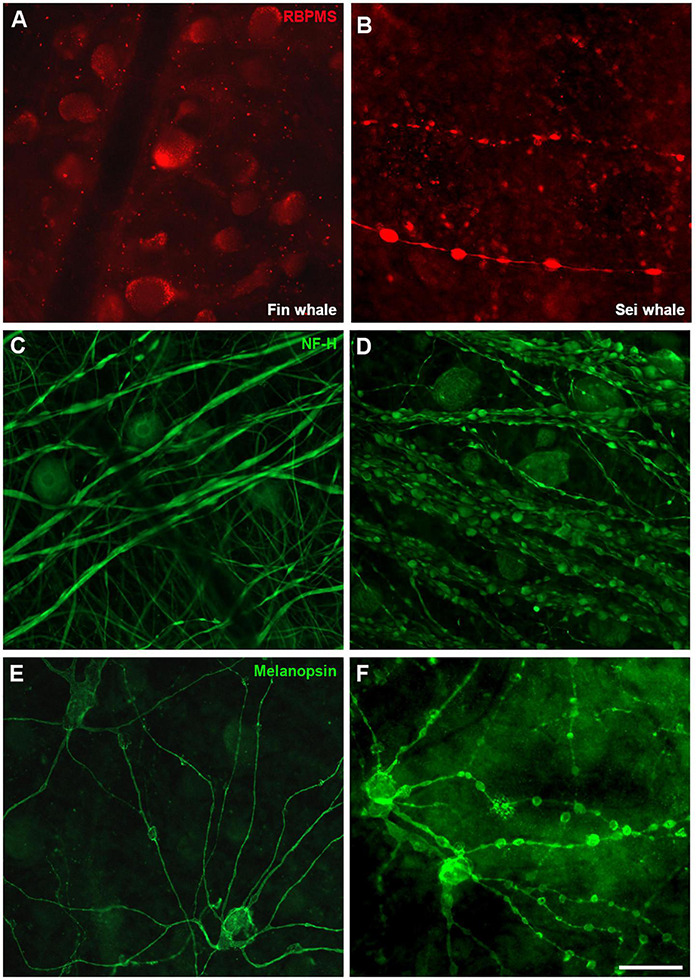
Differences in whale retinas due to tissue conservation. Images of whole mount retinas from fin whale (*Balaenoptera physalus* **A,C,E**) and sei whale (*Balaenoptera borealis* **B,D,F**) fixed 24 and 48 h post-mortem, respectively. The retinas were labelled with antibodies against RBPMS (red, **A,B**), NF-H **(C,D)**, and melanopsin **(E,F)**. Scale bar = 100 μm.

The exact causes of death of the whales were unknown and therefore, signs of neurodegeneration were assessed. The staining of some RGC markers differed in the two whale retinas, whereby the retinas analysed 24 h post-mortem displayed a standard staining pattern while some signs of degeneration were evident in the retinas fixed 48 h post-mortem. Thus, signs of degeneration could be due to the time between the animal death and the time tissues were processed. A distinct distribution of RBPMS was found, shifting from the RGC somas to the axons. In addition, degenerative neurite beading was observed in RGC axons and dendrites with three different markers: RBPMS, NF-H, and melanopsin (Figure 12). The remaining cells of the sei whale retina are similar to those of fin whale (Supplementary Figure 3).

**FIGURE 13:**
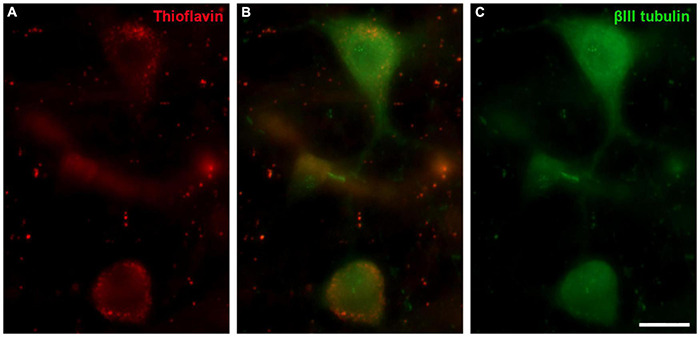
Thioflavin S (ThS) in the fin whale retina. Images of a whole mount whale retina from *Balaenoptera physalus* stained with ThS (red, **A**) and labelled with an antibody against βIII tubulin (green, **B**) to label RGCs. Scale bar = 50 μm.

Thioflavin S (ThS) is commonly used to stain oligomers and plaques of misfolded proteins, as occurs in Alzheimer and Parkinson’s diseases. The well-preserved *B. physalus* retina was stained with ThS and weak punctuate labelling was detected at the edges of some RGCs. This labelling on *B. borealis* retina was not included because the ThS staining could be attributable to the bad conservation of the post-mortem tissue and not to the neurodegeneration state of the whale before death. We confirmed that ThS staining was present in RGCs though it was co-localised with βIII tubulin labelling (Figure 13).

## Discussion

As cetaceans are one of the few mammals that adopt an aquatic lifestyle during evolution, they offer a unique opportunity to study the evolution of the visual system. Our current understanding of the cetacean visual system is rather limited. The study of the extracellular matrix and in particular, the cornea and collagen fibres distribution in relation to their organ size has been performed by our group (Vecino et al., 2021), even though there is abundant evidence that vision plays an important role in their life (Herman, 1990).

Several morphological features of the cetacean eye differ from those of other mammalian eyes. For example, the cetacean eye has well developed extraocular muscles, as well as a thick cornea and sclera to protect against high pressures (Mengual et al., 2015). Collecting the eyes of whales, such as mysticetes (including the genus Balaenoptera), in a sufficiently well-preserved state for histological analysis can be problematic (Lisney and Collin, 2018), although several studies have described morphological characteristics of the cetacean retina, including one of the largest, the fin whale (*Balaenoptera physalus*: Pilleri and Wandeler, 1964). The topographic organisation of RGCs has been analysed in a variety of cetacean species in order to estimate their visual acuity and to compare visual specialisation. Indeed, the density and distribution of RGCs in different whale species has been compared to that in the human retina (Table 2). In addition to the estimation of the total number of RGCs, the number of optic fibres was also quantified in some whales, for example: 420,000 in *B. borealis* (Morgane and Jacobs, 1972); 326,000 in *Balaenoptera acutorostrata* (Jansen and Jansen, 1969); and 252,000 in *Balaenoptera physalis* (Jacobs and Jensen, 1964). The total number of RGCs estimated here, analysing from the center to the periphery of the retina, is similar to that of other odontocetes (as pilot whale) and mysticetes (as grey whale) cetacean species. In addition, the values are similar to the total number of RGCs reported for other artiodactyls as the river hippopotamus (approximately 243,000). But the retinas of other terrestrial artiodactyls, such as the giraffe, the camel, and the water buffalo have 1 million or more RGCs. Conversely, some odontocete cetaceans as the Amazonian River dolphin and the tucuxi, have much lower numbers (approximately 15,000 and 45,000, respectively; Lisney and Collin, 2018). On the other hand, the low RGC density found in the fin whale could be explained by the large very size of their retina. In addition, the use of different markers may influence the differences found in respect to other cetaceans.

The distribution of RGCs, the number of optic fibres, the retinal resolving power, the field of best vision and the direction of the visual axis appear to be similar in most marine cetaceans. However, the density of the RGCs and the retinal resolving power are lower in cetaceans than in terrestrial mammals. Though the vision of cetaceans is suitable to recognise motion and contrast, other sensory modalities are also used to detect objects, such as hearing due to their acute auditory capacity (Murayama et al., 1992; Murayama and Somiya, 1998). Although RGC densities are low, two specialised areas of high cell density are apparent in the vast majority of cetacean species (Lisney and Collin, 2018). These areas appear to provide higher visual resolution and may be considered as the areas of best vision. In addition, the position of these areas correlates with the shape of the pupil (Mass, 1996). The shape, size, and density of RGCs in these areas are associated with the ecology of the species (Hughes, 1977). Another typical feature of the cetacean retina is the presence of RGCs named giant cells, the cell body reaches up to 75 μm in diameter, and cells are separated by wide intercellular spaces. These cells might help detect contrast and movement in the depths of the ocean (Dawson et al., 1982; Mengual et al., 2015). In conclusion, cetaceans do not have prominent visual streaks but rather small areas of peak RGC density, although they do have some huge RGCs with large dendrites and is consistent with the need to function in a very low light intensity environment. Thus, these features have ecological implications, implying that cetaceans and terrestrial mammals have distinct visual sensitivity and spatial resolution (Mass and Supin, 1997; Mass et al., 2013; Mengual et al., 2015).

Cetaceans have a relatively low RGC density compared to land mammals (Figure 3), even in the high density areas of the retina (Mengual et al., 2015). Irrespective of the brain or body size, the absolute number of neurons is a better predictor of cognitive ability (Herculano-Houzel, 2011). Thus, despite their large size, the retinal resolving power in cetaceans can be generally weaker than that of terrestrial mammals due primarily to their low neurons density (Murayama et al., 1992; Murayama and Somiya, 1998). However, most RGCs are larger in cetaceans than in terrestrial mammals, which has ecological implications given the low light intensity of the cetacean environment. These giant RGCs tend to have larger diameter axons to conduct the visual information to longer distances due to the body length. In addition they might help detect contrasts and movement in the ocean’s depths (Mengual et al., 2015). For animals with a large body size like whales, rapid transmission of nerve pulses through the thick axons of large RGCs is important (Dawson et al., 1982). Thus, it has been suggested that the giant RGC-axon systems in the whale retina may be similar to the “Y” cell-axon system (large cell body, broad radiating dendritic tree, and large axon) in terrestrial mammals (Dawson et al., 1982; Mass and Supin, 1997).

Regarding RGC markers, whale RGCs were stained with antibodies against RBPMS, NFs, Brn3a, and βIII tubulin. The NF distribution is similar between whale RGCs and terrestrial mammals, and although not all RGCs express each different type of NF, at least two NF subtypes are detected in a substantial proportion of RGCs. In the porcine and human retina, at least one of each of the three NF subtypes is present in all RGCs, NF-H, and NF-M are distributed widely within all RGC soma and dendrites, whereas NF-L is more restricted to the perinuclear area (Ruiz-Ederra et al., 2004). By contrast, all NFs were distributed widely within the RGC soma in the whale retinas, and NF-H and NF-M were more intensely labelled in the perinuclear area of some cells (Figure 4). The analysis of NFs in cetaceans is important because NFs make up the bulk of the intra-axonal volume in a large axon. The NFs are determinant in generating normal axonal diameters, which is important for normal neuronal activity given that the speed of transmission of the electrical signal along the axon is directly proportional to its diameter: the bigger the diameter, the faster the impulse travels (Lobsiger and Cleveland, 2009). In addition, NFs are more abundant in large RGCs (Ruiz-Ederra et al., 2004) and thus, NFs are crucial for the activity of giant RGCs in whales. Furthermore, while Brn3a is a nuclear marker that serves as a reliable and efficient marker to identify and quantify RGCs (Nadal-Nicolas et al., 2009), Brn3a was not only found in the nucleus but it also spread to the soma in the whale retina.

A small proportion of RGCs express melanopsin, between 1 and 3% in land mammals, and these are ipRGCs (intrinsically photosensitive). These ipRGCs are dedicated to non-image-forming visual functions, including the timing of circadian rhythms and the pupillary light reflex. In the whale, ipRGCs were more abundant in the centre of the retina, diminishing toward the periphery (Figure 5). By contrast, ipRGCs are distributed widely throughout the human retina, although a higher density has been observed in the perifoveal area, and a fewer ipRGCs were found in the vicinity of the optic nerve and in the peripheral retina (Lax et al., 2019). Six types of ipRGC (M1 to M6) have been identified, based on morphological and physiological features, and their dendrite morphology. The majority of ipRGCs are of the M1, M2, or M3 subtypes, and they are the easiest to identify as they express more melanopsin. By contrast, the M4, M5, and M6 ipRGCs express very small quantities melanopsin and they are very difficult to identify by standard immunohistochemical techniques (Lax et al., 2019; Vidal-Villegas et al., 2021). Here, M1, M2, and M3 melanopsin ipRGCs were identified in the whale retina, although they were mostly M2. However, the number of M1 cells is higher than that of M2 and M3 cells in the rodent retina, and displaced M1 (M1d, M1 cells with their soma located in the inner nuclear rather than the ganglion cell layer) represent a small group of ipRGCs (Lax et al., 2019). Conversely, M1d is the predominant subtype in the human retina, accounting for about half of all ipRGCs (Ortuno-Lizaran et al., 2018). This difference between species may be due to the different roles of the ipRGCs subtypes. M1 ipRGCs project to approximately 15 brain targets not involved in image forming activities, and M1 cells predominantly project to the suprachiasmatic nucleus (SCN) to control circadian photoentrainment but also to the shell of the olivary pretectal nucleus (OPN) to control the pupillary light reflex and to other mood-regulating regions. By contrast, M2 ipRGCs send few projections to the SCN and they project more strongly to the OPN. In addition, this subtype projects to more traditionally visual areas like the superior colliculus (Schmidt et al., 2011; Lazzerini Ospri et al., 2017). The M2 subtype is predominant in cetaceans, which means that control of the pupillary light reflex is more important to them than controlling the circadian cycle, as in land mammals where M1 subtype is predominant. This is consistent with the fact that they live deep in the ocean where there is barely any light, even during the day.

Surprisingly, ipRGCs were not labelled with classic RGC molecular markers (Figure 6). For instance, Brn3a was not expressed by ipRGCs in the mouse retina. Brn3a positive cells project to visual brain centres, supporting the view that melanopsin positive cells are involved exclusively in the non-image forming aspects of vision (Jain et al., 2012). This might explain why Brn3a and melanopsin labelling does not co-localise in the whale retina. In addition, rodent ipRGCs express tubulin but not NFs (Vugler et al., 2008), yet βIII tubulin was not expressed either by ipRGCs in the whale retina. Other ipRGCs melanopsin subtype, M4, has been described to express NF-H or SMI-32 (Schmidt et al., 2014), however, this cell subtype was not identified in the whale retina. IpRGCs are a very complex RGC subtype, and the evolutionary significance of their molecular profile is at present unclear. However, it can be hypothesised that ipRGCs do not express the classical markers of RGCs because they do not fulfil visual functions and they project to different regions of the brain than other RGCs.

Most terrestrial mammals have colour vision based on two spectrally different visual pigments located in two types of cone photoreceptors (M/L and S opsins). Using specific antibodies, we demonstrate an absence of cone opsin in the whale retinas studied here (Figure 7), lack of labelling may be due to species specificity of the antibodies used. However, bipolar cell and amacrine immunofluorescence indicate that visual signal transmission is maintained (Figures 8, 9). Studies using electroretinograms in other species, also confirms that their all-rod retinas possess both ON and OFF bipolar cell pathways that are functional (Collin et al., 2009). Previous studies demonstrated rod monochromacy in some cetaceans and several mutations in the opsin gene sequence have been reported in cetaceans, suggesting the evolutionary complete or partial loss of cone cell function in the retina, while non-photosensitive cones are maintained (Schweikert et al., 2016). Refined histological and advanced microscopy techniques revealed two cone morphologies in cetacean retinas that are not traditional morphologic cone structures, a consequence of genetic modifications influenced by environmental selection pressure (Smith et al., 2021). The discovery of rod monochromacy in whales provides an opportunity to investigate the effects of an evolutionary loss of cone photoreceptors on retinal organisation. Rod photoreceptors are more sensitive detectors of light than cones and they are capable of detecting single photons (Field and Rieke, 2002). Thus, rod-based vision provides better underwater vision in conditions where light intensity is low and light is scattered with increasing depth (Smith et al., 2021). In deep-diving species, rod dominance and rapid dark adaptation in particular are traits that indicate these marine mammals use vision primarily in low light levels, where colour vision may be of secondary importance (Peichl et al., 2001).

While whales have adapted to dim light environments and are rod monochromats, the role of ipRGCs could be very important and is not completely understood. Amino acid sequence alignments of cetacean and terrestrial melanopsins reveal very few non-conserved amino acid substitutions, which would result in significant divergence away from this typical absorption maximum. However, the cetacean and land mammalian melanopsins diverge at the carboxy tail, specifically at the sites of phosphorylation that are involved in pigment inactivation (Fasick and Robinson, 2016). Hence, it can be hypothesised that the melanopsin expressed in the retina of rod monochromat cetaceans will have a carboxyl tail that results in slow deactivation kinetics. This slow deactivation of melanopsin will allow these animals to maintain prolonged pupil constriction when they are exposed to bright lights, thereby protecting rod photoreceptors from photo-bleaching (Fasick et al., 2019), and highlighting the importance of the pupillary reflex in cetaceans.

Little is known about the glial cells in the cetacean, with three types of astrocytes identified in the whale brain with distinct thicknesses and lengths of their processes (Pritz-Hohmeier et al., 1994). There were considerably more astroglia in the cetacean optic nerve than in land mammals, with astrocyte processes occupying a higher proportion of the nerve surface and apparently ensheathing every single RGC axon. This high astroglial content in the cetacean nerve could be due to the highly developed metabolic support to CNS neurons required to sustain nervous activity during anaerobic and energy-demanding tasks like prolonged apnoea (Mazzatenta et al., 2001). Surprisingly, there is a punctuate pattern of astrocytes in the whale retina and the identification of individual astrocytes was difficult (Figure 2). Moreover, on the blood vessels, the astroglial network seems to be less complex than in other mammal retinas, probably due to the large size of the blood vessels.

Other glial cells in these cetacean retinas appear to be more robust than in land mammal retinas, like Müller cells, for review (Vecino et al., 2016). However, there are considerable morphological variations among related species, both within an individual retina and among different vertebrate groups (Willbold and Layer, 1998). In addition, while the microglia in the whale retina are larger and more diffused, brain microglia seem to be similarly ramified as in other mammals (Pritz-Hohmeier et al., 1994). The large size of these cells (Figures 10, 11) in whales could be related to the large size of their neurons, such as RGCs, and with the large dimensions of the retina. However, more studies will be needed to fully understand glial cells in the cetacean CNS.

Finally, some signs of degeneration were evident in the whale retinas analysed here (Figure 12), although the causes of the whales’ death were unknown. Some abnormalities were observed, such as a differential distribution of RBPMS in the RGCs. RBPMS was localised to RGC axons rather than RGC somas; this shift in the location of the RBPMS has been observed in other mammalian species as a sign of degeneration (Pereiro et al., 2020). In addition, degenerative neurite beading with focal bead-like swellings on dendrites and axons represents the accumulation of vesicular cargo and transport proteins due to impaired transport, often a neuropathological sign (Takeuchi et al., 2005). However, this neurite beading was observed on RGCs of *B. borealis* probably due to the lag on the fixation and the subsequent degeneration of the tissue.

Thioflavin staining was performed in the retina of the best-preserved whale, *Balaenoptera physalus*, in order to identify if there was any sign of degeneration. In different neurodegenerative diseases, distinct sets of proteins may misfold and form insoluble fibrillar aggregates to which thioflavin binds and emits fluorescence (Arad et al., 2020). The staining found in this study (Figure 13) present a weak labelling mainly in the large RGCs, with a punctuate pattern, and it did not seem to be standard thioflavin staining: a mesh of stained fibrils, a central dense core surrounded by either a corona or compact plaques, with no surrounding material (Bussiere et al., 2004). Thus, although this labelling was not pathological, it could be an early sign of neurodegeneration that may be related to the animal’s death.

## Conclusion

In conclusion, various differences exist between land mammal and whale retinas, such as the low RGC density, the presence of giant RGCs, and the rod-monochromatic vision. While some of the particular characteristics of these cetacean retinas have been studied poorly, their well-developed melanopsin-positive RGC network and characteristic glial cells shown here are features that could be responsible for the special visual perception of these cetaceans.

## Data Availability Statement

The original contributions presented in the study are included in the article/Supplementary Material, further inquiries can be directed to the corresponding authors.

## Ethics Statement

Ethical review and approval was not required for the animal study because the eyes were collected from beached whales and they were studied post-mortem.

## Author Contributions

NR and XP: formal analysis. EV: funding acquisition, project administration, and supervision. NR: methodology and writing—original draft of the manuscript. NR, XP, and EV: writing—review and editing, contributed to manuscript revision, read, and approved the submitted version.

## Conflict of Interest

The authors declare that the research was conducted in the absence of any commercial or financial relationships that could be construed as a potential conflict of interest.

## Publisher’s Note

All claims expressed in this article are solely those of the authors and do not necessarily represent those of their affiliated organizations, or those of the publisher, the editors and the reviewers. Any product that may be evaluated in this article, or claim that may be made by its manufacturer, is not guaranteed or endorsed by the publisher.

## Supplementary Material

Supplementary Figure 1 Distribution of RGCs and ipRGCs across the fin whale retina. Images of a large area of a whole mount fin whale retina labelled with antibodies against NF-H (red) and NH-HP (green, **A**) and melanopsin (green, **B**). Scale bar = 250 μm.

Supplementary Figure 2 M1, M2, and M3 subtypes of melanopsin positive cells in the whale retina. Representative Z-stack images of melanopsin positive RGCs (green) in the whole mount whale retina. The M1 cell with its soma (filled arrowhead) located in the ganglion cell layer (GCL) and with dendrites stratified (unfilled arrowhead) in the outermost (OFF) layer (S1) of the inner plexiform layer (IPL) **(A)**. The M2 cell with its cell body (filled arrowhead) in the GCL and dendrites stratified (unfilled arrowhead) in the innermost (ON) layer of the IPL (S5) **(B)**. The M3 cell with its cell body (filled arrowhead) in the GCL and with dendrites stratified (unfilled arrowhead) in both the S5 and S1 **(C)**. The first photo of each group is the closest to the GCL and the rest of the images were taken every 2 μm in the direction of the inner nuclear layer (IPL), thus, the last photo is the closest to the inner nuclear layer (INL). Scale bar = 100 μm.

Supplementary Figure 3 Retinal cells in the sei whale retina. Representative pictures of neuron and glial cells of the sei whale (*Balaenoptera borealis*). Although this retina has signs of neurodegeneration, it was useful to analyse RGCs and ipRGCs and to verify that the morphology and distribution of the rest of the cells were similar to that of the fin whale. The retina was labelled with antibodies against rhodopsin (red, **A**) to identify rods, S opsin (green, **B**) and M/L opsin (green, **C**) to identify cones, PKC-α (red, **D**) to identify bipolar cells, GFAP (green, **E**) to identify astrocytes, and vimentin (red, **F**) to identify Müller cells. In addition, images of the microglia labelled with antibody against Iba-1 (red) were taken close to the ganglion cell layer **(G)** and to the inner nuclear layer **(H)** in the whole mount retina. The nuclei were stained with DAPI (blue): ONL, outer nuclear layer; OPL, outer plexiform layer; INL, inner nuclear layer; IPL, inner nuclear layer; GCL, ganglion cell layer. Scale bars = 50 μm.
