# Supplementary figures and images for: Immunohistochemical Characterisation of the Whale Retina
Source: Front Neuroanat. 2022 Feb 4;16:813369. doi: 10.3389/fnana.2022.813369 (PMC8856181; doi:10.3389/fnana.2022.813369)

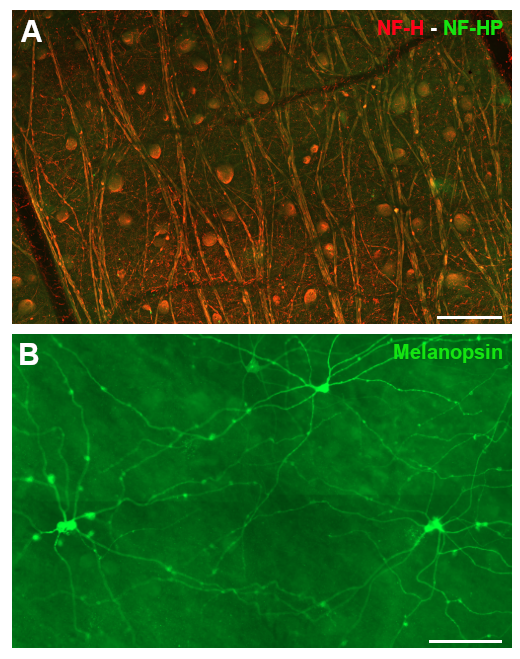

Supplement: Supplementary Figure 1 — Distribution of RGCs and ipRGCs across the fin whale retina. Images of a large area of a whole mount fin whale retina labelled with antibodies against NF-H (red) and NH-HP (green, A) and melanopsin (green, B). Scale bar = 250 μm. [file Image_1.TIFF]

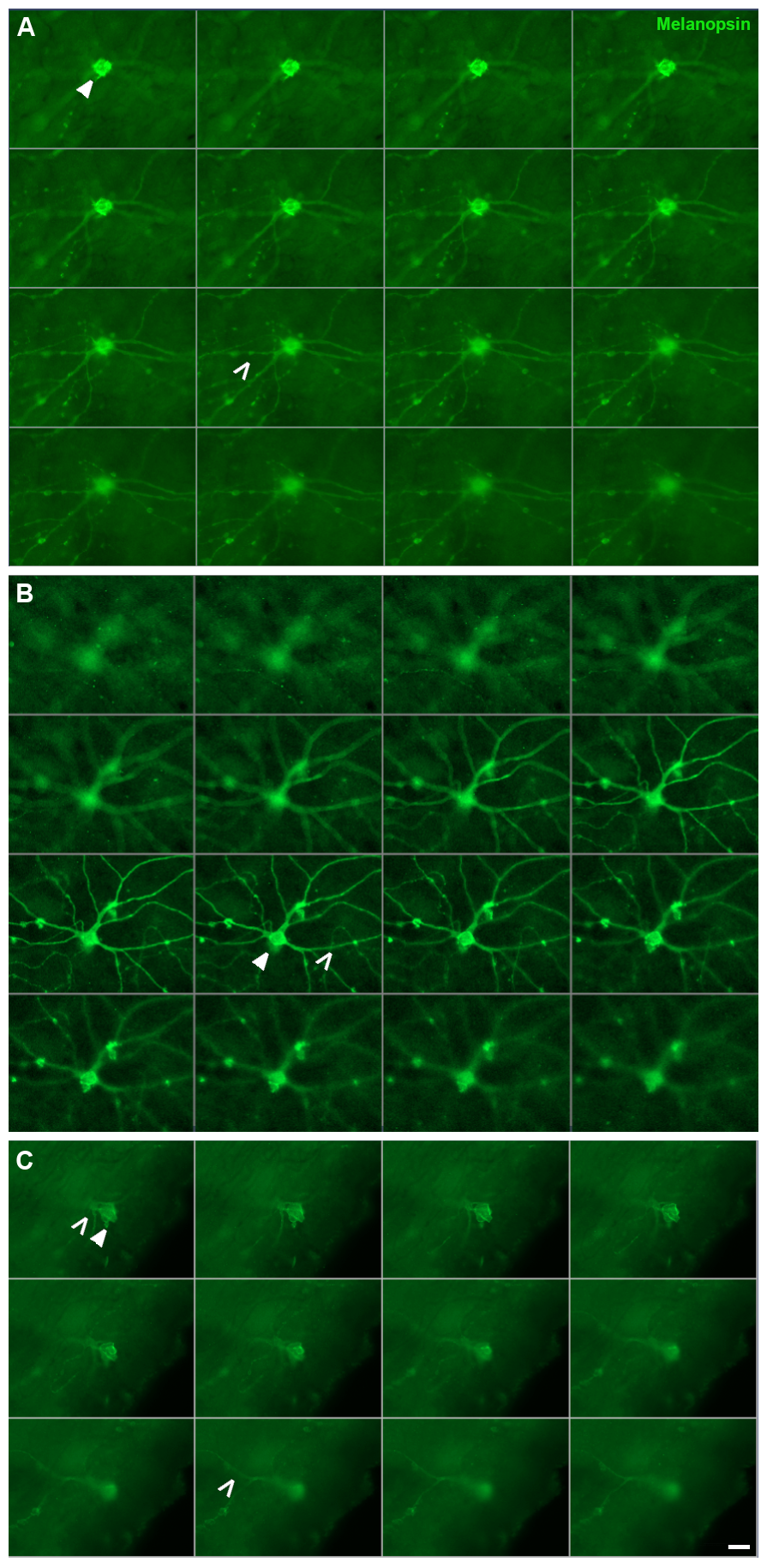

Supplement: Supplementary Figure 2 — M1, M2, and M3 subtypes of melanopsin positive cells in the whale retina. Representative Z-stack images of melanopsin positive RGCs (green) in the whole mount whale retina. The M1 cell with its soma (filled arrowhead) located in the ganglion cell layer (GCL) and with dendrites stratified (unfilled arrowhead) in the outermost (OFF) layer (S1) of the inner plexiform layer (IPL) (A). The M2 cell with its cell body (filled arrowhead) in the GCL and dendrites stratified (unfilled arrowhead) in the innermost (ON) layer of the IPL (S5) (B). The M3 cell with its cell body (filled arrowhead) in the GCL and with dendrites stratified (unfilled arrowhead) in both the S5 and S1 (C). The first photo of each group is the closest to the GCL and the rest of the images were taken every 2 μm in the direction of the inner nuclear layer (IPL), thus, the last photo is the closest to the inner nuclear layer (INL). Scale bar = 100 μm. [file Image_2.TIFF]

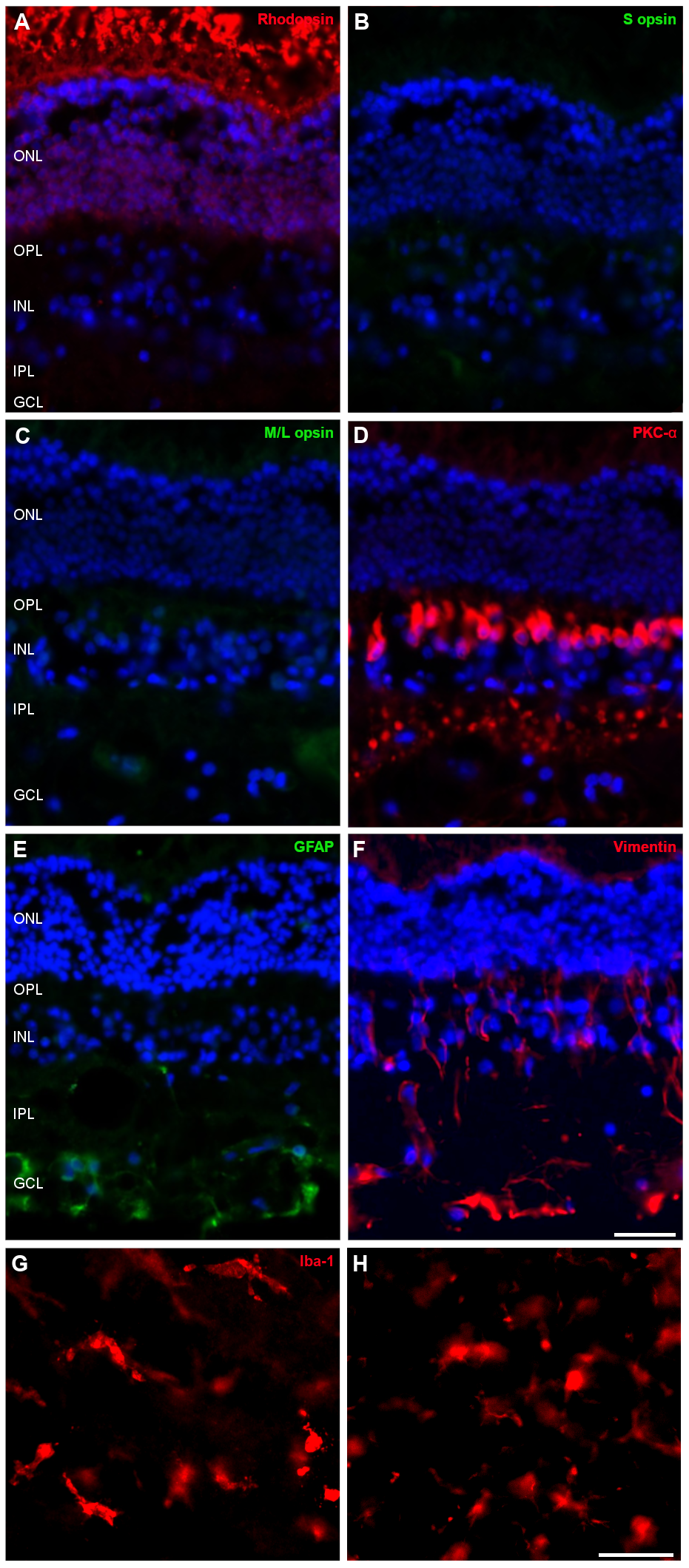

Supplement: Supplementary Figure 3 — Retinal cells in the sei whale retina. Representative pictures of neuron and glial cells of the sei whale (Balaenoptera borealis). Although this retina has signs of neurodegeneration, it was useful to analyse RGCs and ipRGCs and to verify that the morphology and distribution of the rest of the cells were similar to that of the fin whale. The retina was labelled with antibodies against rhodopsin (red, A) to identify rods, S opsin (green, B) and M/L opsin (green, C) to identify cones, PKC-α (red, D) to identify bipolar cells, GFAP (green, E) to identify astrocytes, and vimentin (red, F) to identify Müller cells. In addition, images of the microglia labelled with antibody against Iba-1 (red) were taken close to the ganglion cell layer (G) and to the inner nuclear layer (H) in the whole mount retina. The nuclei were stained with DAPI (blue): ONL, outer nuclear layer; OPL, outer plexiform layer; INL, inner nuclear layer; IPL, inner nuclear layer; GCL, ganglion cell layer. Scale bars = 50 μm. [file Image_3.TIFF]
